# Human liver stem cells and derived extracellular vesicles improve recovery in a murine model of acute kidney injury

**DOI:** 10.1186/scrt514

**Published:** 2014-11-10

**Authors:** Maria Beatriz Herrera Sanchez, Stefania Bruno, Cristina Grange, Marta Tapparo, Vincenzo Cantaluppi, Ciro Tetta, Giovanni Camussi

**Affiliations:** Translational Center for Regenerative Medicine and Molecular Biotechnology Center, University of Torino, Via Nizza 52, 10126 Torino, Italy; Department of Molecular Biotechnology and Health Science, University of Torino, Via Nizza 52, 10126 Torino, Italy; Department of Medical Sciences, University of Torino, Corso Dogliotti 14, 10126 Torino, Italy; EMEA LA Medical Board, Fresenius Medical Care, Bad Homburg, Germany

## Abstract

**Introduction:**

Several cellular sources of stem cells have been tested in the attempt to yield innovative interventions in acute kidney injury (AKI). Human liver stem cells (HLSCs) are cells isolated from the normal adult human liver which are gaining attention for their therapeutic potential. In the present study, we investigated whether HLSCs and the derived extracellular vesicles may promote tubular regeneration after AKI induced by glycerol injection in severe-combined immune-deficient mice.

**Methods:**

HLSCs were expanded and conditioned medium (CM) and extracellular vesicles (EVs) were purified. HLSCs and their bioproducts were tested in a model of AKI induced by intra-muscle glycerol injection. Renal function and morphology were evaluated five days after induction of damage. The effect of EVs on proliferation and apoptosis of murine renal tubular cells was tested *in vitro*.

**Results:**

We found that intravenous injection of 3.5×10^5^ HLSCs into mice three days after induction of AKI significantly improved functional and morphological recovery. The injection of HLSCs decreased creatinine and urea, as well as hyaline cast formation, tubular necrosis and enhanced *in vivo* tubular cell proliferation. The effect of soluble factors release by HLSCs in the regenerative processes was also studied. CM produced by HLSCs, mimicked the effect of the cells. However, depletion of EVs significantly reduced the functional and morphological recovery of CM. Moreover, we found that purified HLSC-derived EVs ameliorated renal function and morphology in a manner comparable to the cells. *In vitro* HLSC-derived EVs were shown to stimulate proliferation and inhibit apoptosis of murine renal tubular cells.

**Conclusions:**

These results indicate that HLSCs increase recovery after AKI. EVs are the main component of HLSC-derived CM capable of promoting regeneration in experimental AKI.

## Introduction

We recently identified and characterized adult human liver stem cells (HLSCs) [1]. HLSCs expressed markers characteristic of the mesenchymal lineage together with albumin and alpha-fetoprotein, suggesting a partial hepatic commitment but lack of hematopoietic stem markers. HLSCs were also able to undergo multiple *in vitro* differentiations. *In vivo*, we demonstrated the therapeutic potential of HLSCs in several experimental models of liver injury [1, 2]. We found that HLSCs contribute to regeneration of the liver parenchyma in acetaminophen induced-injury [1] and significantly attenuate mouse mortality in a model of fulminant liver failure in severe-combined immune-deficient (SCID) mice [2]. It has been recently suggested that the biological effects of stem cells are mediated by paracrine factors acting on neighboring cells [3, 4]. Indeed, Bi *et al*. showed that conditioned medium (CM) derived from mesenchymal stem cells (MSCs) diminishes the apoptosis of tubular cells and limits renal injury [5] in a model of acute kidney injury (AKI) induced by cisplatin. Similarly, in a model of fulminant hepatic injury induced by D-galactosamine and lipopolysaccharide, we found that CM from HLSCs mimicked the beneficial effect of cells [2]. In addition to soluble factors, extracellular vesicles (EVs) released from stem cells have been shown to contribute to renal recovery after AKI [6, 7].

A seminal work of De Broe *et al*. [8] demonstrated that cell-derived circular plasma-membrane fragments retained an enzymatic activity similar to that of the originating cells. Subsequent studies demonstrated that EVs play a critical role as mediators of cell-to-cell communication [9]. Bruno *et al*. [6] showed that EVs released from MSCs promoted the recovery from AKI, stimulated a proliferative program in tubular epithelial cells and reduced lethality in SCID mice treated with cisplatin [10]. Similarly, EVs-derived from MSCs reduced injury and stimulated recovery in a model of renal ischemia/reperfusion [7]. We found that, HLSC-derived EVs induced *in vitro* proliferation and apoptosis resistance of human and rat hepatocytes and accelerated *in vivo* the morphological and functional recovery of the liver in a model of 70% hepatectomy in rats [11].

The therapeutic potential of HLSCs in diseased organs different from the liver has not yet been evaluated. The aim of the present study was to investigate whether HLSCs, HLSC-derived CM and HLSC-derived EVs contribute to tubular regeneration in AKI induced in mice by intramuscular injection of glycerol in SCID.

## Methods

### Isolation and characterization of HLSCs

HLSCs were isolated from human cryopreserved normal adult hepatocytes (Lonza, Basel, Switzerland). The isolation, culture and characterization of HLSCs were performed as previously described [1]. Briefly, hepatocytes were initially cultured for two weeks in Hepatozyme-SFM medium. After the two weeks, most hepatocytes died, and then the medium was substituted by α-MEM/EBM-1 (3:1) (Invitrogen, Carlsbad, CA, USA) media supplemented with l-glutamine (5 mM), Hepes (12 mM, pH 7.4), penicillin (50 IU/ml), streptomycin (50 μg/ml) (all from Sigma, St. Louis, MO, USA) and fetal calf serum (FCS) (10%) (Invitrogen). Cells were expanded and characterized. HLSCs expressed the mesenchymal stem cell markers but not the hematopoietic and endothelial markers as described [1]. HLSCs were also positive for human albumin and alpha-fetoprotein and for the resident stem cells markers vimentin and nestin, and were negative for CD34, CD117 and cytokeratin 19 oval cell markers [1]. HLSCs also expressed the embryonic stem cell markers nanog, Oct4, Sox2 and SSEA4 [2]. HLSCs were shown to undergo osteogenic, endothelial and hepatic differentiation under appropriate culture conditions [1].

### Isolation of HLSC-derived EVs

EVs were obtained from supernatants of HLSCs (2 × 10^6^ cells/T75) cultured for 24 hours in Roswell Park Memorial Institute medium (RPMI) deprived of FCS. The viability of HLSCs at the time of EV collection was 97% to 99% as detected by trypan blue exclusion and no apoptotic cells were detectable by TUNEL assay.

After removal of cell debris and apoptotic bodies by two centrifugations at 3,000 g for 20 minutes, EVs were purified by two hours ultracentrifugation (100,000 g) at 4°C (Beckman Coulter Optima L-90 K, Fullerton, CA, USA). EVs were used fresh or stored at -80°C after re-suspension in RPMI supplemented with 5% dimethyl sulfoxide (DMSO). No difference in biological activity was observed between fresh and stored EVs.

To trace EVs by fluorescent microscopy, EVs were labeled with 1 μM Dil dye (Molecular Probes, Life Technology, NY, NY, USA) during ultracentrifugation [12]. After labeling, EVs were washed by ultracentrifugation at 100,000 g for one hour at 4°C. EV pellets were suspended in (D)MEM.

As control, EVs were inactivated by sonication (15 minutes at 60 kHz) and heating (40°C) to disrupt the membrane (iEV).

Analysis of the size distribution of EVs was performed using NanoSight LM10 (NanoSight Ltd, Minton Park, UK) [10].

### Characterization of HLSC-derived EVs

EVs were characterized by cytofluorimetric analysis using fluorescein isothiocyanate (FITC), phycoerythrin (PE) or allophycocyanin (APC) conjugated antibodies against CD73, CD44, CD105, CD90, CD107, CD63, CD29, CD81, CD146 and HLA-class I. Conjugated mouse non-immune isotypic immunoglobulin G (IgG) (Miltenyi Biotec, Bergisch Gladbach, Germany) was used as control. Briefly, EVs (0.15 × 10^9^ particles) were incubated for 15 minutes at 4°C with antibodies, then diluted 1 to 3 and immediately acquired as previously described [12, 13]. Samples were acquired using Guava easyCyte Flow Cytometer (Millipore, Billerica, MA, USA) and analyzed with InCyte software. We also performed fluorescence-activated cell sorting (FACS) analysis after absorption on beads. Briefly, EVs (10 μg) were incubated for 30 minutes to overnight in ice with 5 μl of latex beads (Aldeyde/sulphate LATEX 4MM, Invitrogen), then washed in PBS supplemented with 100 mM glycine and incubated for 30 minutes with the antibodies described above.

Protein content of the EV preparations was quantified by the Bradford method (Bio-Rad, Hercules, CA, USA).

Protein samples were separated by 4% to 15% gradient sodium dodecyl sulfate–polyacrylamide gel electrophoresis and subjected to immunoblotting with antibodies CD63, CD81, Alix (Santa Cruz Biotechnology, Santa Cruz CA, USA) and Hsp90 (Cell Signaling Technologies, Danvers, MA, USA). The protein bands were visualized with an enhanced chemiluminescence (ECL) detection kit and ChemiDoc™ XRS + System (BioRad). Cell and EV lysates were loaded at a concentration of 10 μg/well.

### Preparation of HLSC-derived CM

HLSC-derived CM was produced as previously described [2]. Briefly, HLSC-CM was obtained from 20 × 10^5^ cells/T75 cultured for 24 hours in RPMI deprived of FCS as for the collection of EVs. After two centrifugations at 3,000 g for 20 minutes to remove cell debris, cell-free CM were concentrated 25-fold by centrifugation at 2,700 g for 75 minutes, using Ultra-PL 3 ultrafiltration units (Amicon-Ultra; Millipore) with a 3-kDa molecular weight cutoff. A total of 250 μL of CM was obtained.

To evaluate the contribution of EVs in the biological effect of CM, in select experiments CM was depleted of EVs by ultracentrifugation (100,000 g for five hours); depletion was verified by NanoSight analyses, as described below.

### Isolation and culture of murine tubular epithelial cells and *in vitro*experiments

Kidneys were obtained from healthy female C57 mice. Murine tubular epithelial cells (mTEC) were isolated, cultured and characterized for the presence of tubular markers and for the absence of endothelial and glomerular markers as previously described by Bruno *et al*. [6]. To determine the incorporation efficacy of HLSC-derived EVs, we incubated Dil labeled EVs, purified from 25,000 HLSCs, with 25,000 mTEC for five hours. The up-take of EVs was analyzed using confocal microscopy (Zeiss LSM 5 Pascal, Carl Zeiss, Oberkochen, Germany).

For proliferation experiments, mTECs were seeded at 4,000 cells/well into 96-well plates in 100 μl/well of (D)MEM low glucose with 2% FCS and in the presence of CM deprived of EVs, EVs and iEV derived from 8,000 HLSCs. DNA synthesis was detected as incorporation of 5-bromo-2′-deoxy-uridine (BrdU) into the cellular DNA after 48 hours of culture (Roche Applied Science, Mannheim, Germany).

For apoptosis experiments, mTECs were seeded at 25,000 cells/well into 24-well plates in (D)MEM low glucose without FCS in the presence of CM deprived of EVs, EVs and iEV derived from 50,000 HLSCs.

Cell death analysis was performed using the Muse_Annexin V & Dead Cell Assay (Millipore) after 48 hours. The assay was performed according to the manufacturer’s protocols.

### SCID mice model of AKI

Animal studies were conducted in accordance with the National Institutes of Health Guide for the Care and Use of Laboratory Animals. The protocol entitled: ‘Experimental models of renal regenerative therapies’ was approved on 22 November 2012 by the Committee on Bioethics of the University of Torino, Italy.

To evaluate the ability of HLSCs, HLSC-derived EVs or HLSC-derived CM to improve kidney injury, we induced AKI by intra-muscle injection of glycerol (Sigma) in SCID mice as previously described [6]. Mice were anesthetized with isoflurane and then injected with 50% glycerol in water, 8 ml/kg, half of the dose injected into each muscle of the inferior hind limbs. Glycerol induced myolysis and hemolysis, thereby exposing the kidney to large amounts of myoglobin and hemoglobin [6]. The peak of tubular injury was observed three days after glycerol injection. At this time, mice received different treatments. The following groups were studied: group 1, AKI mice intravenously (iv) injected with vehicle alone (n = 18); group 2, healthy mice iv injected with vehicle alone (n = 6); group 3, AKI mice iv injected with 0.75 × 10^5^ (n = 4) or 3.5 × 10^5^ (n = 7) HLSCs; group 4, AKI mice iv injected with concentrated CM HLSC-derived non EV depleted obtained from 3.5 × 10^5^ HLSCs (n = 9), 10 × 10^5^ HLSCs (n = 5), or 20 × 10^5^ HLSCs (n = 10); group 5, AKI mice iv injected with CM HLSC-derived EV depleted (n = 11) obtained from 20 × 10^5^ HLSCs; group 6, AKI mice iv injected with EVs produced by 3.5 × 10^5^ HLSCs (n = 9; EV1) or with EVs derived from 10 × 10^5^ HLSCs (n = 4; EV2). Sonicated EVs produced by 3.5 × 10^5^ HLSCs were iv injected in AKI mice (group 7, n = 3).

Finally, Dil labeled EV1 were injected in AKI (group 8, n = 3) and in healthy mice (group 9, n = 3).

The iv injections were performed in 120 μL in the tail vein. In all experiments, cells cultured in T75 flasks until the 2 to 6 passage were detached by trypsin (0.5% w/v), washed, and resuspended in PBS.

To quantify proliferation of renal cells, BrdU (100 mg/Kg) was injected intraperitoneally for two consecutive days before the mice were killed.

### Renal function

Blood samples for measurement of blood urea nitrogen (BUN) and plasma creatinine were collected five days after glycerol-induced AKI. Plasma creatinine was measured using a colorimetric microplate assay based on the Jaffe reaction (Quantichrom Creatinine Assay, BioAssay Systems, Hayward, CA, USA). BUN was measured by direct quantification of plasma urea with a colorimetric assay kit according to the manufacturer’s protocol (Quantichrom Urea Assay, BioAssay Systems).

### Morphological studies

For renal histology, 5 μm-thick paraffin kidney sections were routinely stained with hematoxylin and eosin (H&E, Merck, Darmstadt, Germany) for microscopic examination.

Luminal hyaline casts and cell necrosis (denudation of tubular basement membrane) were assessed in non-overlapping fields (10 for each section) using a 40× objective (high power field, HPF). The number of casts and tubular profiles showing necrosis were recorded in a single-blind fashion.

Immunohistochemistry for detection of proliferation of tubular cells was performed as described previously [6]. Kidney sections were subjected to antigen retrieval, and slides were blocked and labeled with 1:25 dilution of monoclonal anti BrdU antibody (Dako Cytomation, Milan, Italy), or 1:400 of monoclonal anti-proliferating cell nuclear antigen (PCNA, Santa Cruz Biotechnology). Immunoperoxidase staining was performed using 1:300 dilution of anti-mouse horseradish peroxidase (HRP, Pierce, Rockford, IL, USA). Scoring for BrdU- and PCNA-positive cells was carried out by counting the number of positive nuclei per HPF (40×) in 10 randomly chosen sections of kidney cortex.

Confocal microscopy analysis was performed on frozen sections for localization of Dil-labeled EVs in kidneys of healthy and AKI mice. Sections were blocked and labeled with rabbit anti laminin (Sigma) (1:100 dilution). Omission of the primary antibodies or substitution with non immune rabbit IgG was used as control. Alexa Fluor 488 anti-rabbit (Molecular Probes) was used as secondary antibody.

### Statistical analysis

Results are expressed as mean ± standard deviation (SD). Statistical analysis was performed by using the *t* test, analysis of variance (ANOVA) with Newmann-Keuls or ANOVA with Dunnet’s multicomparison tests when appropriate. A *P* value of <0.05 was considered significant.

## Results

### HLSCs favor AKI recovery

**Figure 1:**
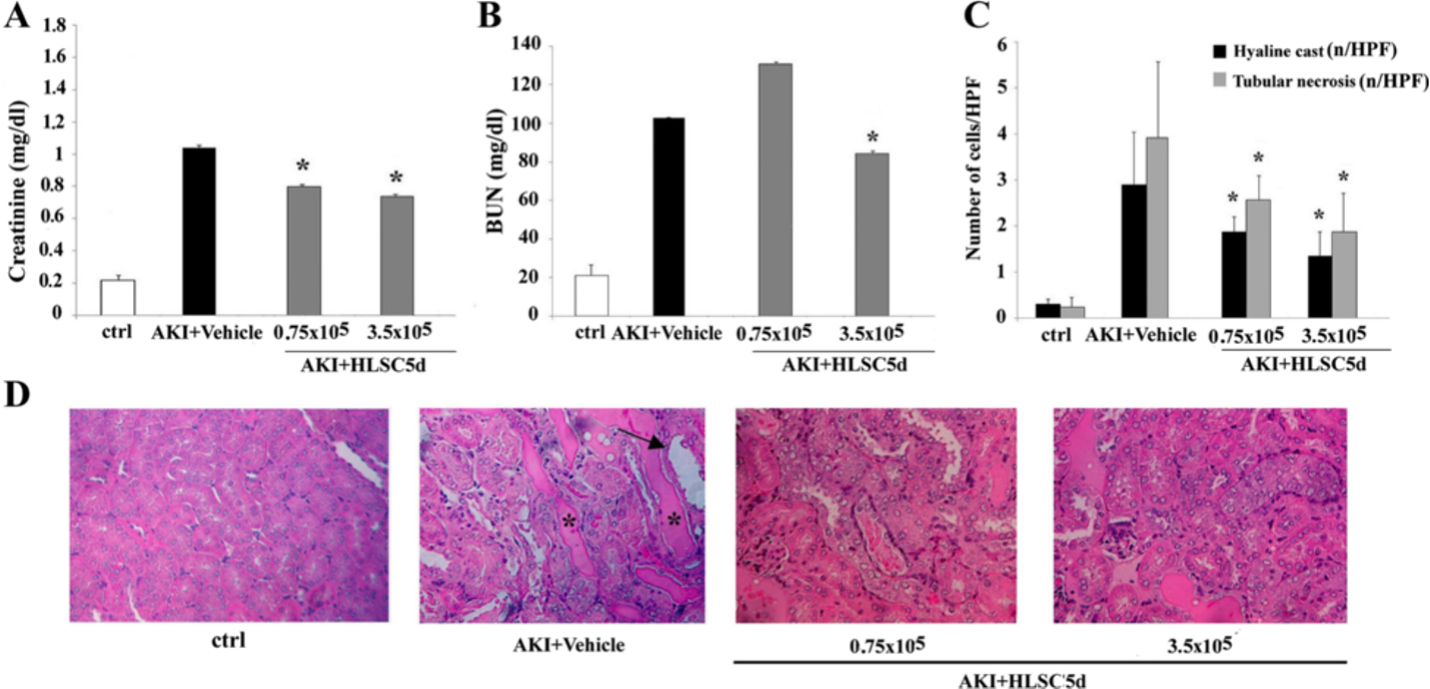
**Effects of intravenous injection of HLSCs in AKI mice. (A)** and **(B)** Creatinine and blood urea nitrogen (BUN) values on day 5 after glycerol administration in mice injected with vehicle alone, with HLSCs (0.75 × 10^5^; 3.5 × 10^5^) and in healthy (ctrl) SCID mice. Data are expressed as mean ± SD; ANOVA with Dunnet’s multicomparison test: *P <0.05 HLSC-treated (0.75 × 10^5^; 3.5 × 10^5^) AKI mice versus vehicle-treated AKI mice. **(C)** Effect of HLSCs injection on renal morphology at day 5 after AKI induction. The number of hyaline casts and tubular necrosis observed under high power (original magnification: ×400) is expressed as mean ± SD. ANOVA with Dunnet’s multicomparison test: *P <0.05 HLSC-treated (0.75 × 10^5^; 3.5 × 10^5^) AKI mice versus vehicle-treated AKI mice. **(D)** Representative micrographs of renal histology of healthy SCID mice (ctrl) and of AKI mice treated with vehicle alone or with different quantities of HLSCs (0.75 × 10^5^; 3.5 × 10^5^) and sacrificed at day 5. Original magnification: ×200. The typical aspect of hyaline cast formations and tubular necrosis are, respectively, shown by asterisks and head arrows. AKI, acute kidney injury; ANOVA, analysis of variance; HLSCs, human liver stem cells; SD, standard deviation.

To determine whether HLSCs were capable of favoring renal regeneration, we induced AKI in SCID mice with an intramuscular injection of hypertonic glycerol. At day 3 after injury, 0.75 × 10^5^ and 3.5 × 10^5^ HLSCs were injected in AKI mice that were sacrificed at day 5. In AKI mice significant increases in plasma levels of creatinine and BUN were observed at day 3 (Figure 1A and B). Mice injected with 0.75 × 10^5^ and 3.5 × 10^5^ HLSCs exhibited, at day 5 (48 hours after treatment), a significant reduction of creatinine levels (Figure 1A and B) at both cells concentrations, whereas a significant reduction of BUN was observed only with 3.5 × 10^5^ HLSCs (Figure 1A and B). Histological analysis confirmed the functional results (Figure 1C and D). The morphological alterations observed in SCID mice with AKI included tubular hyaline cast formation and tubular necrosis (Figure 1D). AKI mice treated with 0.75 × 10^5^ and 3.5 × 10^5^ HLSCs had a significantly lower number of hyaline cast formations and lower number of necrotic tubules compared with AKI mice treated with vehicle alone (Figure 1C and D).

**Figure 2:**
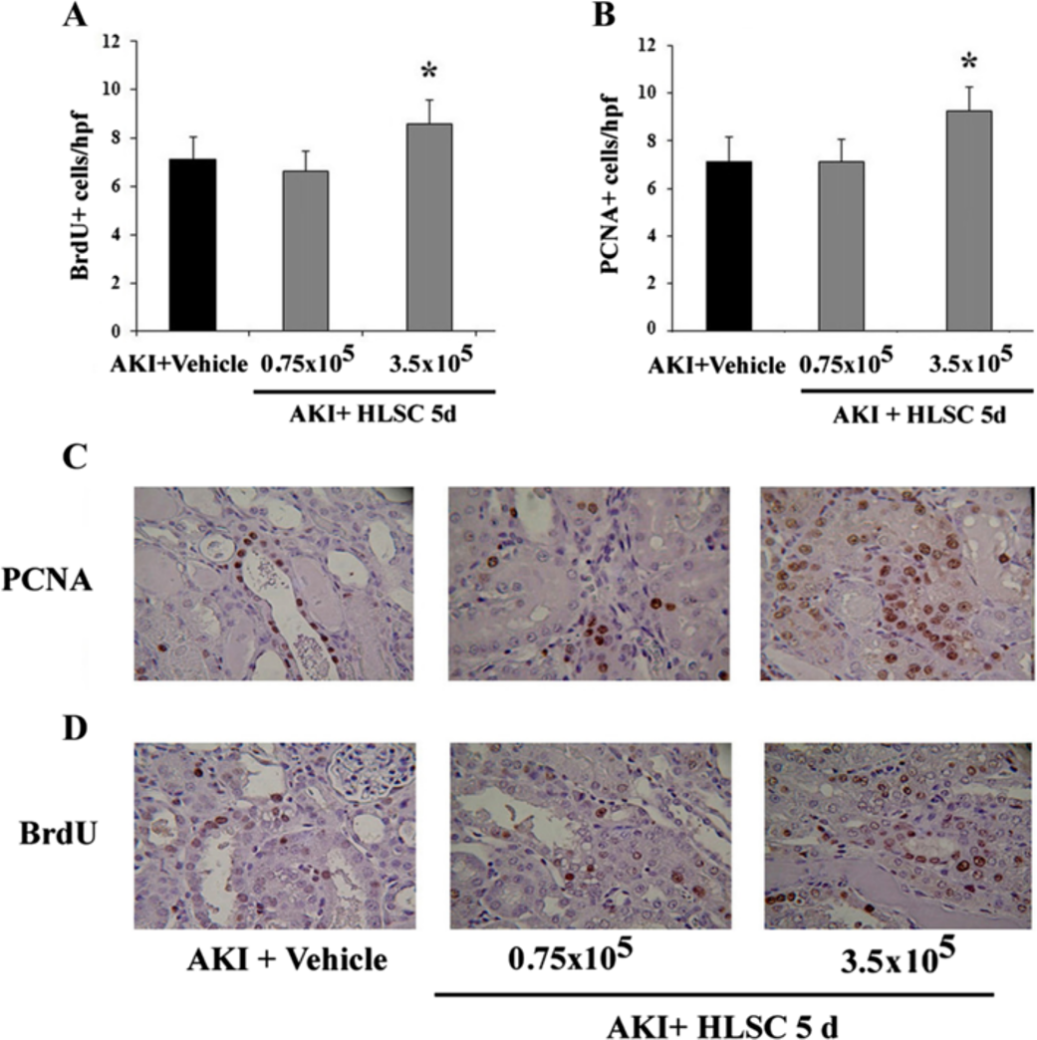
**Renal cell proliferation in AKI mice treated with HLSCs.** Quantification of BrdU **(A)** and PCNA **(B)** positive cells/hpf was performed in renal sections of AKI mice treated with vehicle alone or with different quantities of HLSCs and sacrificed after five days. BrdU was injected intraperitoneally for two consecutive days before mice were killed. Data are expressed as mean ± SD. ANOVA with Dunnet’s multicomparison test was performed: * *P* <0.05 HLSC-treated AKI mice versus vehicle-treated AKI mice. Representative micrographs of PCNA **(C)** or BrdU **(D)** uptake staining performed on sections of kidneys five days after glycerol treatment (two days after vehicle or HLSC injection). Original magnification: ×400. AKI, acute kidney injury; ANOVA, analysis of variance; BrdU, 5-bromo-2′-deoxy-uridine; HLSCs, human liver stem cells; PCNA, proliferating cell nuclear antigen; SD, standard deviation.

The effect of HLSC treatment on tubular cell proliferation was investigated by studying the expression of PCNA and the uptake of BrdU by tubular cells in treated or untreated AKI mice (Figure 2). Proliferation of tubular cells was significantly increased in mice treated with 3.5 × 10^5^ HLSCs with respect to AKI mice treated with vehicle alone (Figure 2). The lower dose of HLSCs (0.75 × 10^5^) failed to stimulate tubular proliferation.

### CM reproduced the protective effect of HLSCs

**Figure 3:**
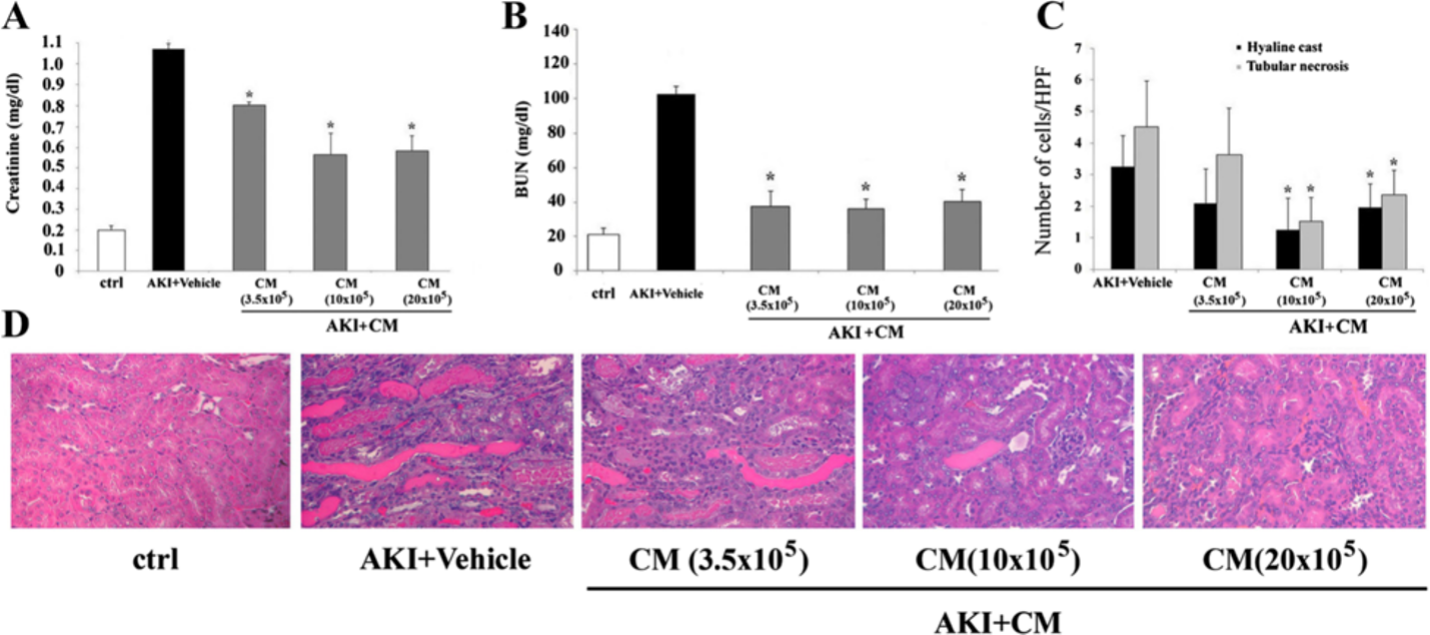
**Effects of intravenous injection of HLSC-derived CM into AKI mice.** Creatinine **(A)** and BUN **(B)** were measured in mice treated with vehicle alone and in HLSC-derived CM-treated mice five days after glycerol injection and in healthy control (ctrl) mice. Data are expressed as mean ± SD; ANOVA with Dunnet’s multicomparison test: *P <0.05 HLSC-derived CM-treated AKI mice versus vehicle-treated AKI mice. **(C)** Comparison of HLSC-derived CM injections on tubular morphology at day 5 after AKI induction. Data are expressed as mean ± SD of hyaline cast and necrotic tubules observed under high power (original magnification: ×400). ANOVA with Dunnet’s multicomparison test: *P <0.05 HLSC-derived CM-treated AKI mice versus vehicle-treated AKI mice. **(D)** Representative micrographs of renal histology of healthy SCID mice and of AKI mice treated with vehicle alone or injected intravenously with concentrated CM derived from different quantities of HLSCs (CM (3.5 × 10^5^), CM (10 × 10^5^), CM (20 × 10^5^)) (original magnification: ×200). AKI, acute kidney injury; ANOVA, analysis of variance; BUN, blood urea nitrogen; CM, conditioned medium; HLSCs, human liver stem cells; SCID, severe–combined immune-deficient; SD, standard deviation.

In order to study the effect of paracrine factors produced by HLSCs, different preparations of concentrated CM derived from 3.5 × 10^5^, 10 × 10^5^ and 20 × 10^5^ HLSCs were injected in AKI mice (Figure 3). At day 5 after glycerol injection, treatments with CM resulted in significant decreases of creatinine and BUN levels (Figure 3A and B) compared to mice treated with vehicle alone. Also, tubular injury improved in mice treated with CM (Figure 3C and D). In particular, hyaline tubular casts and the degree of necrosis were reduced with respect to mice given vehicle alone. CM produced by 3.5 × 10^5^ HLSCs failed to attenuate tubular injury (Figure 3C and D).

**Figure 4:**
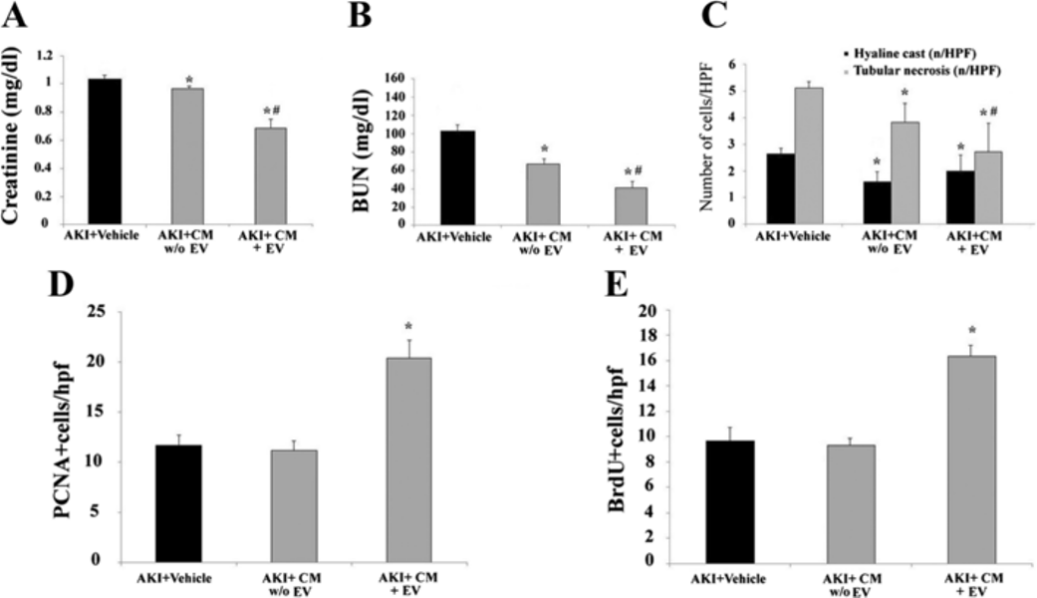
**Effect of concentrated CM depleted of EVs on AKI recovery.** Creatinine **(A)** and BUN **(B)** were measured in AKI mice treated with vehicle alone or with CM depleted (AKI + CM w/o EVs) or not of EVs (AKI + CM + EVs) five days after glycerol injection. Data are expressed as mean ± SD; Analysis of variance with Newmann-Keuls multicomparison test was performed; *P <0.05 CM-treated AKI mice versus vehicle-treated AKI mice; ^#^ *P* <0.05 CM + EVs- treated AKI mice versus CM w/o EVs-treated AKI mice. **(C)** Comparison of tubular morphology in AKI mice injected with HLSC-CM depleted or not of EVs. Data are expressed as mean ± SD of hyaline cast and tubular necrosis observed under high power (original magnification: ×400). Analysis of variance with Newmann-Keuls multicomparison test *P <0.05 CM = treated AKI mice versus vehicle-treated AKI mice, ^#^ *P* <0.05 CM + EV-treated AKI mice versus CM w/o EV-treated AKI mice. Quantification of PCNA **(D)** and BrdU **(E)** positive cells/hpf in AKI mice treated with vehicle alone or CM with or without (w/o) EVs and CM + EVs and sacrificed after five days. BrdU was injected intraperitoneally for two successive days before mice were killed. Data are expressed as mean ± SD. ANOVA with Dunnet’s multicomparison test was performed: * *P* <0.05 CM + EV-treated AKI mice versus vehicle-treated AKI mice. AKI, acute kidney injury; ANOVA, analysis of variance; BrdU, 5-bromo-2′-deoxy-uridine; BUN, blood urea nitrogen; CM, conditioned medium; EV, extracellular vesicles; HLSCs, human liver stem cells; hpf, high power field; PCNA, proliferating cell nuclear antigen; SD, standard deviation; w/o, without.

Concentrated CM contained 71.0 ± 7.0 × 10^9^ EVs/ml as detected by Nanosight. In order to study the contribution of EVs in the regenerative properties of CM, EV-depleted CM obtained from 20 × 10^5^ cells was used. Depletion of EVs did not abrogate but significantly reduced the functional and morphological recovery (Figure 4A-C). More evident was the effect on proliferation. Only EV-containing CM was able to stimulate proliferation as detected by PCNA expression and BrdU incorporation (Figure 4D and E).

### HLSC-derived EV characterization and *in vitro*effects

**Figure 5:**
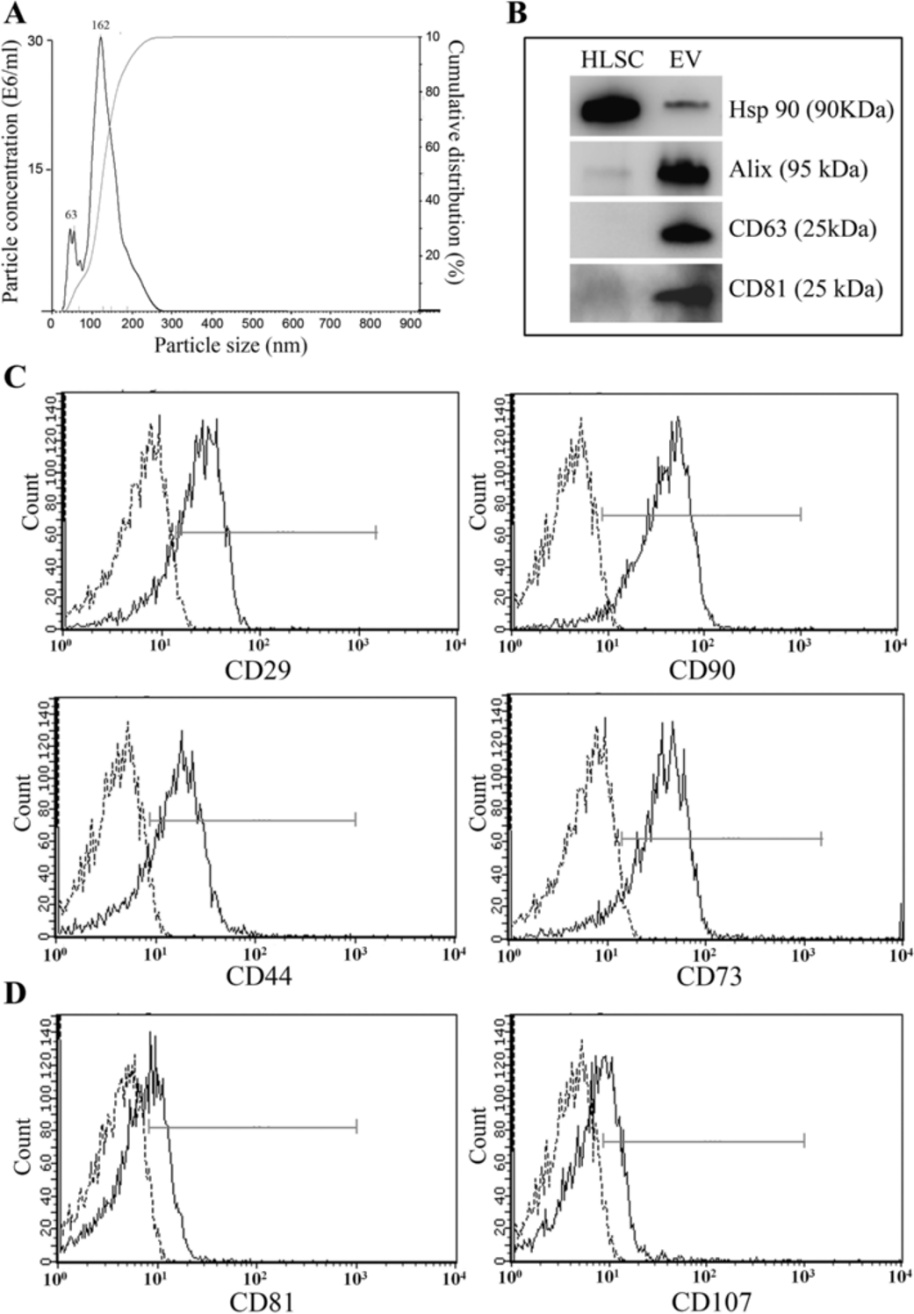
**Characterization of HLSC-derived EVs surface markers and NTA analyses. (A)** Nanosizer analysis of purified HLSC-derived EVs: curve 1 shows the relationship between particle distribution (left Y axis) and particle size (X axis); curve 2 shows the correlation between cumulative percentage distribution of particles (percentile in right Y axis) and particle size (X axis). Mean size and particle concentration values were calculated by Nanoparticle Tracking Analysis (NTA). **(B)** Representative Western blot analysis of exosomal markers CD63, CD81, Alix and Hsp90 expressed by HLSCs and derived EV (10 μg protein/well). **(C-D)** Representative FACS analyses of the expression of classical mesenchymal **(C)** and exosomal markers **(D)** by HLSC-derived EVs. Dot lines indicate the isotopic controls. Three different EV preparations were tested with similar results. EVs, extracellular vesicles; FACS, fluorescence-activated cell sorting; HLSC, human liver stem cell.

By nanoparticle tracking analysis (NTA) the EV population appeared heterogeneous in size distribution (Figure 5). The mean size of EVs was 174 ± 64 nm, with the presence of small EVs with a diameter of about 60 nm (Figure 5A). The expression of some classical exosomal markers (CD81, CD63, Alix and Hsp90) was detected by Western blot analysis (Figure 5B).

Guava FACS analyses showed the presence of several mesenchymal surface markers characteristic of HLSCs, such as CD29, CD90, CD44 and CD73 and of exosomal markers CD81 and CD107 (Figure 5C and D). Similar results were obtained by FACS analyses performed on vesicles pre-absorbed on beads (not shown).

**Figure 6:**
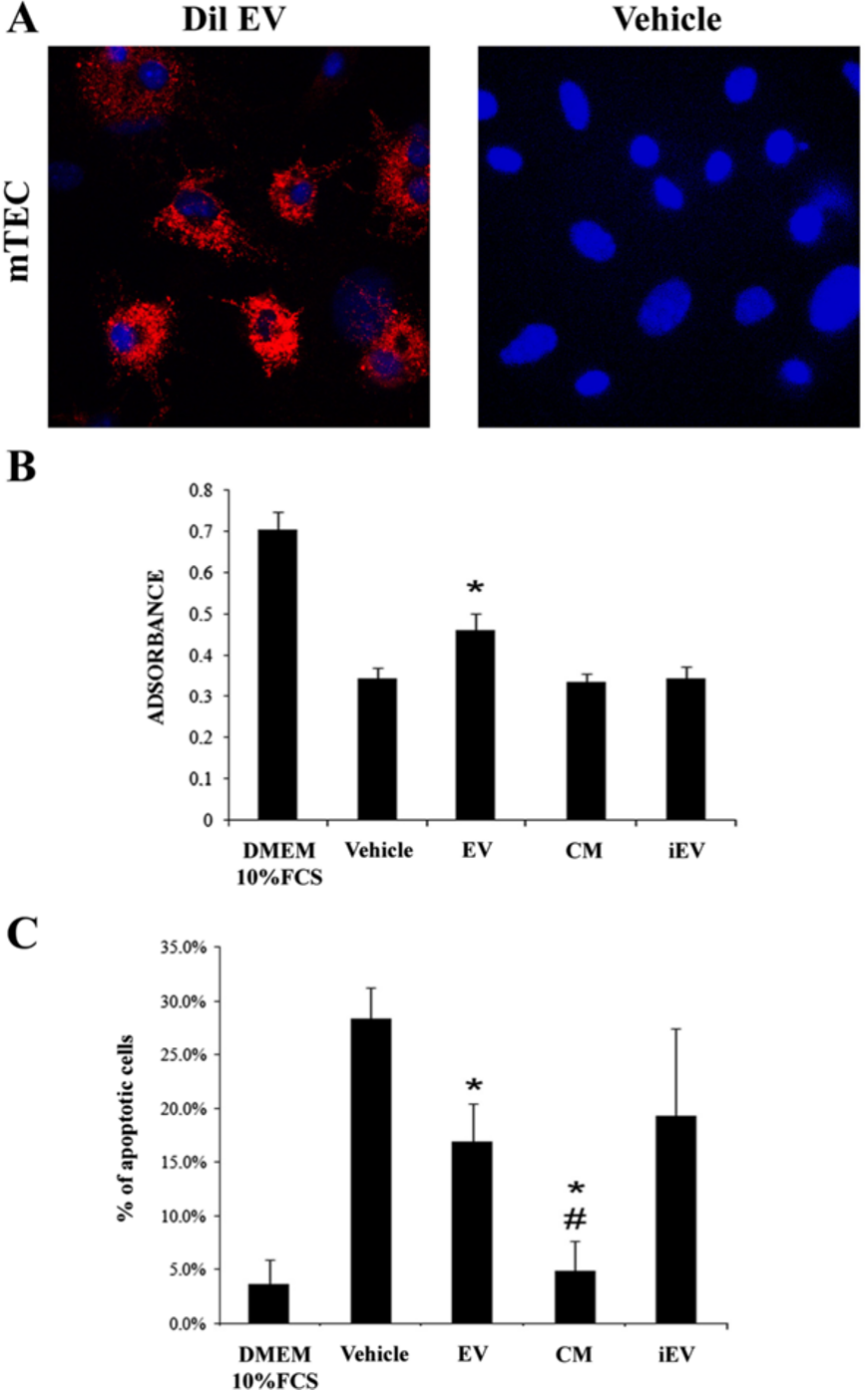
**Up-take of EVs by murine tubular epithelial cells. (A)** Representative micrograph of incorporation of EVs collected from HLSCs stained with Dil (red) by mTEC after five hours of incubation. Original magnification: ×630. Nuclei were stained with Hoechst. Three experiments were performed with similar results. **(B)** Quantification of proliferation rate by BrdU incorporation assay. mTEC (4,000 cells/well) were treated with vehicle, EV, CM and iEV derived from 8,000 HLSCs. Experiments were conducted in triplicate. Data are expressed as mean ± SD; ANOVA with Dunnet’s multicomparison test was performed: *P <0.05 EV versus vehicle. **(C)** Evaluation of apoptosis of mTEC (25,000 cells/well) treated with vehicle, CM, EV and iEV derived from 50,000 HLSCs by Muse Annexin V & Dead Cell Assay. Data are expressed as mean ± SD; ANOVA with Newmann-Keuls multicomparison test was performed: *P <0.05 EV and CM versus vehicle; #*P* <0.05 CM versus EV. ANOVA, analysis of variance; BrdU, 5-bromo-2′-deoxy-uridine; CM, conditioned medium; EV, extracellular vesicles; HLSCs, human liver stem cells; iEV, inactivated extracellular vesicles; mTEC, murine tubular epithelial cells; SD, standard deviation.

To evaluate the ability of EV-HLSCs to be incorporated by mTEC, Dil labeled EVs were incubated with the cells. EV-HLSCs were incorporated by mTEC, as observed by confocal microscopy after five hours of incubation at 37°C (Figure 6A).

To understand the possible different effect of CM and EVs derived from HLSCs on cell proliferation and apoptosis, *in vitro* experiments were performed. Incubation of mTECs with EVs promoted significant cell proliferation with respect to control cells incubated with vehicle alone or with CM deprived of EVs or with iEVs (Figure 6B). Moreover, incubation of mTECs with CM completely inhibited apoptosis induced by serum deprivation (Figure 6C), indicating the relevant anti-apoptotic effect of paracrine factors present in CM. EVs also significantly reduced the apoptosis in mTEC but were less effective than CM (Figure 6C).

No stimulation of proliferation and protection from apoptosis was observed with iEVs.

### HLSC-derived EVs mediated the improvement of renal function and morphology in AKI and stimulated tubular cell proliferation

**Figure 7:**
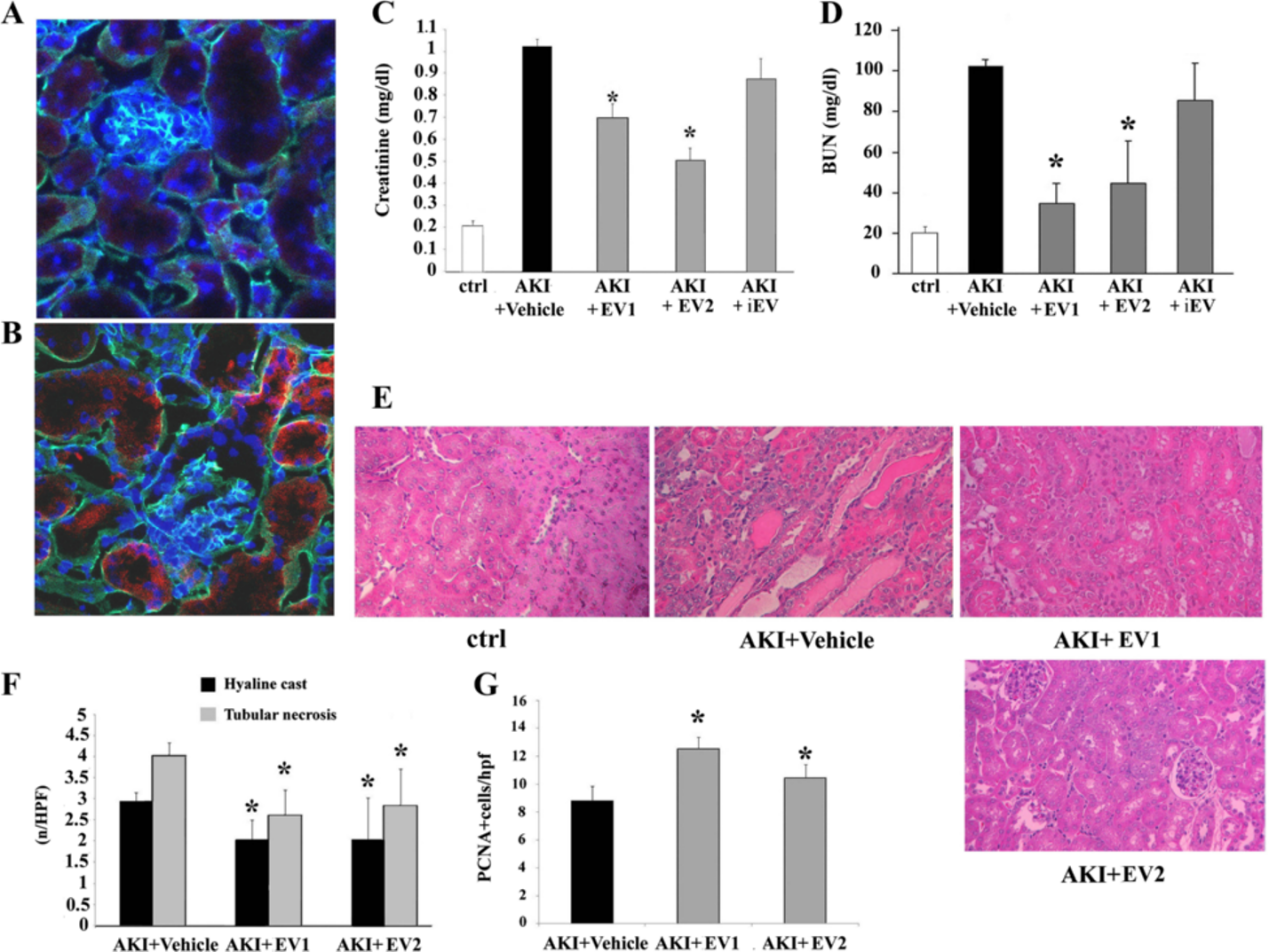
**Effects of intravenous injection of purified EVs released from HLSCs. (A-B)** Representative micrographs of renal tissues of healthy mice **(A)** and of AKI mice **(B)** treated with Dil labeled EVs (red), five hours after the injection. Original magnification: ×630. Nuclei were stained in blue with Hoechst and laminin in green. **(C)** Creatinine and **(D)** BUN values on day 5 after glycerol administration. Data are expressed as mean ± SD; ANOVA with Dunnet’s multicomparison test was performed: *P <0.05 EV-treated AKI mice versus vehicle-treated AKI mice. **(E)** Representative micrographs of renal histology of control healthy mice (ctrl) or AKI mice at day 5 after glycerol administration intravenously injected with vehicle alone (AKI + vehicle) or with EVs derived from 3.5 × 10^5^ HLSCs (EV1) or from 10 × 10^5^ HLSCs (EV2). Original magnification: ×400. **(F)** Comparison of HLSC-derived EV injection on tubular morphology at day 5 after AKI induction. Data are expressed as mean ± SD of hyaline casts and necrotic tubules observed under high power (original magnification: ×400). ANOVA with Dunnet’s multicomparison test was performed: *P <0.05 EV-treated AKI mice versus vehicle-treated AKI mice. **(G)** Quantification of PCNA-positive cells/hpf in AKI mice untreated or treated with HLSC-derived EVs after five days of AKI. Data are expressed as mean ± SD; ANOVA with Dunnet’s multicomparison test: *P <0.05 EV- treated AKI mice versus vehicle- treated AKI mice. AKI, acute kidney injury; ANOVA, analysis of variance; BUN, blood urea nitrogen; CM, conditioned medium; EV, extracellular vesicles; HLSCs, human liver stem cells; hpf, high power field; PCNA, proliferating cell nuclear antigen; SD, standard deviation.

Labeled EVs, produced by 3.5 × 10^5^ HLSCs (EV1), were injected in healthy and AKI mice; five hours after the injection mice were sacrificed and kidneys recovered for analysis. Labeled EVs accumulated preferentially within injured kidneys (Figure 7A and B) and were detectable within tubules and glomeruli. Two concentrations of purified EVs (EV1 = 1.88 ± 0.6 × 10^9^ particles produced by 3.5 × 10^5^ HLSCs; EV2 = 5.53 ± 2.15 × 10^9^ particles produced by 10 × 10^5^ HLSCs) were injected three days after induction of AKI. At day 5 after glycerol injection, EVs significantly decreased creatinine and BUN plasma levels (Figure 7C and D) and improved tubular injury (Figure 7E and F) when compared with vehicle alone. The increased expression of PCNA by tubular cells suggested that EVs stimulated proliferation (Figure 7G).

The injection of iEV did not accelerate the recovery of the tubular damage (Figure 7C and D).

## Discussion

In the present study, we demonstrated that HLSCs favor recovery of glycerol-induced AKI in SCID mice. Moreover, CM of HLSCs mimicked the effect of the cells. EV depletion significantly reduced the healing properties of CM. Purified EVs had the same effect as HLSCs suggesting that HLSC-derived EVs are responsible for the improved renal function.

Several studies demonstrated the beneficial effect of stem cells in the murine experimental model of AKI [14–16]. Many studies focused on the exogenous administration of MSCs because of their regenerative potential and tropism for damaged tissues [15, 17]. The infusion of murine MSCs has been shown to accelerate kidney recovery in cisplatin- or glycerol-induced AKI [14, 15]. In ischemia-reperfusion injury, rat MSCs protected from ischemic acute renal failure through the production of factors with anti-apoptotic and mitogenic activity [18]. The low number of MSCs engrafted in the injured kidney in the face of the strong functional recovery raised the hypothesis that soluble mediators may explain the renoprotective effect of MSCs [19]. It has been suggested that EVs may interact with target cells and transfer functional proteins, micro-RNAs and mRNAs that may modify their phenotype [9, 20]. The biological effect of human MSC-derived EVs has been shown in several xenogenic models suggesting that carried biological molecules are also active in different species [6, 7, 21–24].

In a recent study, Bruno *et al*. [6] documented that EVs produced by MSCs shuttled a specific subset of cellular mRNAs that stimulate survival and proliferation of injured tubular cells. Furthermore, in a lethal model of AKI induced by cisplatin, we found that EVs released by MSCs enhanced survival and that multiple injections of EVs restored normal histology and renal function at day 21 [10].

On the other hand, we found that HLSC paracrine mediators also exert biological activities [2, 11]. We demonstrated that HLSC-derived EVs stimulated proliferation and favored regeneration in a model of 70% hepatectomy in rats by a mechanism involving the transfer of specific mRNA subsets [11]. Moreover, the injection of HLSC-derived CM significantly attenuates mouse mortality in a model of fulminant liver failure induced by D-galactosamine and lipopolysaccharide in SCID mice [2].

In the present study, we found that iv injection of HLSCs at a concentration of 3.5 × 10^5^ cells/mouse in SCID mice with glycerol-induced AKI protected mice from tubular injury and functional impairment. HLSCs promoted histological and functional amelioration of renal damage; moreover, HLSCs stimulated proliferation of tubular epithelial cells. When we injected CM derived from HLSCs in injured SCID mice, we observed an improvement of function and morphological recovery of kidney in comparison with mice treated with vehicle alone. However, when CM was depleted of EVs by ultracentrifugation, CM failed to stimulate tubular proliferation. In contrast, purified EVs reproduced the same biological effect as HLSCs promoting AKI recovery. *In vitro* HLSC-derived EVs stimulated proliferation and inhibited apoptosis of murine renal tubular cells.

## Conclusions

The results of the present study indicate that HLSCs have a healing action in a model of AKI characterized by extensive damage of proximal tubular epithelial cells. HLSC treatment improved renal function and stimulated proliferation of tubular epithelial cells thus favoring recovery. CM mimicked the effect of HLSCs suggesting a paracrine mechanism involving EVs released by these cells. In fact, CM depleted of EVs abated the beneficial effect and purified EVs exhibited the same regenerative effect as HLSCs.

## Abbreviations

(D)MEM: (Dulbecco’s) modified Eagle’s medium
AKI: acute kidney injury
BrdU: 5-bromo-2′-deoxy-uridine
BUN: blood urea nitrogen
CM: conditioned medium
EVs: extracellular vesicles
FACS: fluorescence-activated cell sorting
FCS: fetal calf serum
HLSCs: human liver stem cells
HPF: high power field
iEVs: inactivated extracellular vesicles
MSCs: mesenchymal stem cells
mTEC: murine tubular epithelial cells
NTA: nanoparticle tracking analysis
PBS: phosphate-buffered saline
PCNA: proliferating cell nuclear antigen
SCID: severe-combined immune-deficient mice.
